# (*E*)-2-(5,5-Dimethyl­hexa­hydro­pyrimidin-2-yl)-4-(phenyl­diazen­yl)phenol

**DOI:** 10.1107/S1600536808038877

**Published:** 2008-11-26

**Authors:** Iran Sheikhshoaie, Niaz Monadi, Alireza Abbasi

**Affiliations:** a Chemistry Department, Shahid Bahonar University, Kerman, Iran; b School of Chemistry, University College of Science, University of Tehran, Tehran, Iran

## Abstract

In the title Schiff base, C_18_H_22_N_4_O, the hexa­hydro­pyrimidinyl ring adopts a chair conformation. The dihedral angle between the aromatic rings of the 4-(2-phenyl­diazen­yl)phenol unit is 15.7 (1)°. There is an intra­molecular O—H⋯N hydrogen bond between the hydroxyl group and an N atom of the hexa­hydro­pyimidinyl unit. Inter­molecular N—H⋯O and N—H⋯N hydrogen bonds give rise to a layer structure.

## Related literature

For applications and related structures, see: Farrell *et al.* (2007 ▶).
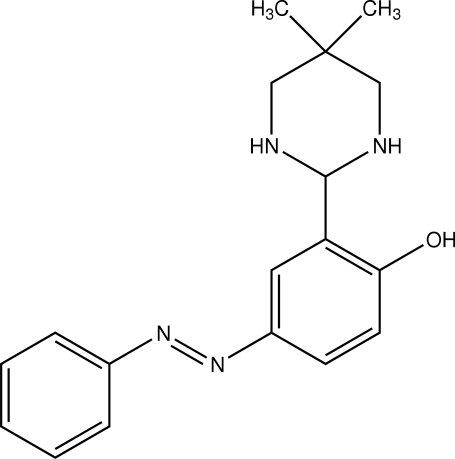


## Experimental

### 

#### Crystal data

C_18_H_22_N_4_O
*M* *_r_* = 310.40
Orthorhombic, 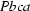

*a* = 9.0287 (9) Å
*b* = 12.0767 (12) Å
*c* = 30.866 (3) Å
*V* = 3365.5 (6) Å^3^
*Z* = 8
Mo *K*α radiation
μ = 0.08 mm^−1^
*T* = 120 (2) K
0.23 × 0.20 × 0.16 mm

#### Data collection

Bruker SMART 1000 CCD area-detector diffractometer
Absorption correction: multi-scan (*SADABS*; Sheldrick, 1996 ▶) *T*_min_ = 0.970, *T*_max_ = 0.987
14483 measured reflections
3134 independent reflections
1574 reflections with *I* > 2σ(*I*)
*R*_int_ = 0.078

#### Refinement

*R*[*F* ^2^ > 2σ(*F* ^2^)] = 0.050
*wR*(*F* ^2^) = 0.114
*S* = 0.81
3134 reflections
234 parameters
H atoms treated by a mixture of independent and constrained refinement
Δρ_max_ = 0.22 e Å^−3^
Δρ_min_ = −0.22 e Å^−3^

### 

Data collection: *SMART* (Bruker, 1998 ▶); cell refinement: *SAINT-Plus* (Bruker, 1998 ▶); data reduction: *SAINT-Plus*; program(s) used to solve structure: *SHELXS97* (Sheldrick, 2008 ▶); program(s) used to refine structure: *SHELXL97* (Sheldrick, 2008 ▶); molecular graphics: *DIAMOND* (Brandenburg, 2001 ▶); software used to prepare material for publication: *PLATON* (Spek, 2003 ▶).

## Supplementary Material

Crystal structure: contains datablocks I, global. DOI: 10.1107/S1600536808038877/ng2518sup1.cif

Structure factors: contains datablocks I. DOI: 10.1107/S1600536808038877/ng2518Isup2.hkl

Additional supplementary materials:  crystallographic information; 3D view; checkCIF report

## Figures and Tables

**Table 1:** Hydrogen-bond geometry (Å, °)

*D*—H⋯*A*	*D*—H	H⋯*A*	*D*⋯*A*	*D*—H⋯*A*
O1—H1*A*⋯N3	1.01 (2)	1.65 (2)	2.584 (2)	152 (2)
N3—H3*A*⋯N4^i^	0.92 (2)	2.30 (2)	3.159 (3)	155 (2)
N4—H4*A*⋯O1^ii^	0.93 (2)	2.18 (2)	3.106 (3)	172 (2)

Symmetry codes: (i) 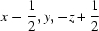; (ii) 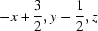.

**Fig. 1.**
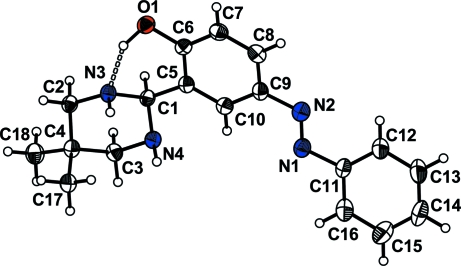
Molecular structure of (I), with 50% probability displacement ellipsoids. H atoms are shown as circles of arbitrary radii.

## supplementary crystallographic information

### Comment

Heterocycles containing nitrogen atoms *e.g.* hexahydropyrimidines have applications in both inorganic and organic chemistry. Hexahydropyrimidines can be easily prepared from condensations of alkyl diamines and aldehydes. Our interest in synthesizing derivatives of these heterocycles was due to their anti-carcinoma, anti-lymphoma, and anti-biotic properties.

The molecular structure of (I) and the atom-numbering scheme are shown in Fig. 1. Two aromatic rings A (C5—C10) and B (C11—C16) show a little deviation from planarity with a dihedral angle of 15.7 (1)*^o^*. Hexahydropyrimidine has a chair conformation. Intramolecular hydrogen bonds are formed between the phenol hydroxyl groups and the nearest N atom in the hexahydropyimidine groups [O—H···N = 2.584 (2) Å]. The packing of the structure is stabilized by relatively strong N—H···O & N—H···N hydrogen bonds (see Tab. 1), and C—H···π contacts [C—H-cetroid = 2.70 Å] between neighboring molecules. No significant π-π interactions are found in the crystal structure.

### Experimental

The title compound was prepared *via* condensation of (*E*)-5-(2-phenyldiazenyl)-2-hydroxybenzaldehyde and 2,2-dimethylpropane-1,3-diamine in 20 ml EtOH:CHCl3. The mixture solution was stirred and refluxed for 3 h. Colorless prismatic-shape crystals were obtained after evaporation of the excess solvent.

### Refinement

Aromatic and methyl H atoms were placed in calculated positions (C—H = 0.93 Å, C—H = 0.96 Å, respectively) and constrained to ride on their parent atoms, with *U*_iso_(H) = 1.2 and 1.5 *U*_eq_(C), respectively. Methylene, hydroxyl and amine H atoms were located in difference density maps and their coordinates were refined freely with *U*_iso_(H) = 1.5 *U*_eq_(C, O & N).

### Crystal data

**Table:**

C_18_H_22_N_4_O	*F*_000_ = 1328
*M**_r_* = 310.40	*D*_x_ = 1.225 Mg m^−^^3^
Orthorhombic, *P**b**c**a*	Mo *K*α radiation λ = 0.71073 Å
Hall symbol: -P 2ac 2ab	Cell parameters from 14483 reflections
*a* = 9.0287 (9) Å	θ = 3–60º
*b* = 12.0767 (12) Å	µ = 0.08 mm^−^^1^
*c* = 30.866 (3) Å	*T* = 120 (2) K
*V* = 3365.5 (6) Å^3^	Prism, colorless
*Z* = 8	0.23 × 0.20 × 0.16 mm

### Data collection

**Table:**

Bruker SMART 1000 CCD area-detector diffractometer	3134 independent reflections
Radiation source: fine-focus sealed tube	1574 reflections with *I* > 2σ(*I*)
Monochromator: graphite	*R*_int_ = 0.078
*T* = 120(2) K	θ_max_ = 25.5º
φ and ω scans	θ_min_ = 2.6º
Absorption correction: multi-scan(SADABS; Sheldrick, 1996)	*h* = −10→10
*T*_min_ = 0.970, *T*_max_ = 0.987	*k* = −14→14
14483 measured reflections	*l* = −32→37

### Refinement

**Table:**

Refinement on *F*^2^	Secondary atom site location: difference Fourier map
Least-squares matrix: full	Hydrogen site location: inferred from neighbouring sites
*R*[*F*^2^ > 2σ(*F*^2^)] = 0.050	H atoms treated by a mixture of independent and constrained refinement
*wR*(*F*^2^) = 0.114	*w* = 1/[σ^2^(*F*_o_^2^) + (0.049*P*)^2^] where *P* = (*F*_o_^2^ + 2*F*_c_^2^)/3
*S* = 0.81	(Δ/σ)_max_ < 0.001
3134 reflections	Δρ_max_ = 0.22 e Å^−^^3^
234 parameters	Δρ_min_ = −0.22 e Å^−^^3^
Primary atom site location: structure-invariant direct methods	Extinction correction: none

### Special details

**Table:**

Geometry. All e.s.d.'s (except the e.s.d. in the dihedral angle between two l.s. planes) are estimated using the full covariance matrix. The cell e.s.d.'s are taken into account individually in the estimation of e.s.d.'s in distances, angles and torsion angles; correlations between e.s.d.'s in cell parameters are only used when they are defined by crystal symmetry. An approximate (isotropic) treatment of cell e.s.d.'s is used for estimating e.s.d.'s involving l.s. planes.
Refinement. Refinement of *F*^2^ against ALL reflections. The weighted *R*-factor *wR* and goodness of fit *S* are based on *F*^2^, conventional *R*-factors *R* are based on *F*, with *F* set to zero for negative *F*^2^. The threshold expression of *F*^2^ > σ(*F*^2^) is used only for calculating *R*-factors(gt) *etc*. and is not relevant to the choice of reflections for refinement. *R*-factors based on *F*^2^ are statistically about twice as large as those based on *F*, and *R*- factors based on ALL data will be even larger.

### Fractional atomic coordinates and isotropic or equivalent isotropic displacement parameters (Å^2^)

**Table:**

	*x*	*y*	*z*	*U*_iso_*/*U*_eq_	
O1	0.69820 (17)	0.17061 (13)	0.26940 (5)	0.0314 (4)	
H1A	0.710 (3)	0.121 (2)	0.2433 (7)	0.047*	
N1	0.9722 (2)	−0.15729 (17)	0.40388 (6)	0.0301 (5)	
N2	0.9051 (2)	−0.06703 (18)	0.41075 (6)	0.0312 (5)	
N3	0.7588 (2)	0.00338 (17)	0.22029 (6)	0.0259 (5)	
H3A	0.692 (3)	−0.0499 (19)	0.2283 (7)	0.039*	
N4	0.9679 (2)	−0.11263 (17)	0.24545 (6)	0.0260 (5)	
H4A	0.911 (3)	−0.1732 (19)	0.2543 (7)	0.039*	
C1	0.8894 (3)	−0.0082 (2)	0.24903 (7)	0.0237 (6)	
H1	0.964 (2)	0.0526 (18)	0.2397 (6)	0.036*	
C2	0.8017 (3)	−0.0160 (2)	0.17456 (7)	0.0261 (6)	
H21	0.876 (3)	0.0485 (19)	0.1681 (7)	0.039*	
H22	0.707 (2)	−0.0077 (18)	0.1561 (7)	0.039*	
C3	1.0059 (3)	−0.1371 (2)	0.20003 (8)	0.0277 (6)	
H31	1.087 (2)	−0.081 (2)	0.1916 (7)	0.042*	
H32	1.048 (2)	−0.212 (2)	0.1986 (7)	0.042*	
C4	0.8774 (2)	−0.1275 (2)	0.16755 (7)	0.0265 (6)	
C5	0.8460 (2)	0.0185 (2)	0.29537 (7)	0.0237 (6)	
C6	0.7534 (2)	0.11069 (19)	0.30298 (7)	0.0244 (6)	
C7	0.7169 (3)	0.1417 (2)	0.34482 (7)	0.0306 (6)	
H7	0.6563	0.2028	0.3495	0.037*	
C8	0.7703 (3)	0.0821 (2)	0.37939 (7)	0.0302 (6)	
H8	0.7471	0.1039	0.4075	0.036*	
C9	0.8586 (2)	−0.0104 (2)	0.37271 (7)	0.0267 (6)	
C10	0.8971 (2)	−0.0407 (2)	0.33038 (7)	0.0261 (6)	
H10	0.9581	−0.1017	0.3259	0.031*	
C11	1.0190 (3)	−0.2133 (2)	0.44232 (7)	0.0295 (6)	
C12	1.0096 (3)	−0.1697 (2)	0.48356 (7)	0.0423 (7)	
H12	0.9718	−0.0989	0.4878	0.051*	
C13	1.0566 (3)	−0.2317 (3)	0.51851 (8)	0.0508 (8)	
H13	1.0503	−0.2024	0.5463	0.061*	
C14	1.1127 (3)	−0.3367 (3)	0.51259 (8)	0.0501 (8)	
H14	1.1445	−0.3778	0.5363	0.060*	
C15	1.1217 (3)	−0.3807 (3)	0.47163 (8)	0.0481 (8)	
H15	1.1587	−0.4518	0.4676	0.058*	
C16	1.0754 (3)	−0.3187 (2)	0.43638 (8)	0.0404 (7)	
H16	1.0822	−0.3481	0.4086	0.049*	
C17	0.7683 (3)	−0.22207 (19)	0.17308 (7)	0.0326 (6)	
H17A	0.7266	−0.2195	0.2017	0.049*	
H17B	0.8186	−0.2913	0.1690	0.049*	
H17C	0.6905	−0.2152	0.1520	0.049*	
C18	0.9416 (3)	−0.1313 (2)	0.12165 (7)	0.0371 (7)	
H18A	0.8627	−0.1258	0.1009	0.056*	
H18B	0.9936	−0.1998	0.1175	0.056*	
H18C	1.0089	−0.0705	0.1177	0.056*	

### Atomic displacement parameters (Å^2^)

**Table:**

	*U*^11^	*U*^22^	*U*^33^	*U*^12^	*U*^13^	*U*^23^
O1	0.0319 (10)	0.0309 (11)	0.0312 (10)	0.0044 (8)	0.0002 (8)	0.0017 (8)
N1	0.0269 (12)	0.0339 (13)	0.0294 (12)	−0.0031 (11)	−0.0025 (9)	0.0034 (10)
N2	0.0262 (12)	0.0355 (13)	0.0320 (12)	−0.0021 (11)	−0.0005 (10)	0.0029 (10)
N3	0.0220 (11)	0.0308 (13)	0.0249 (11)	−0.0014 (10)	−0.0025 (9)	0.0016 (10)
N4	0.0241 (12)	0.0288 (12)	0.0250 (11)	−0.0012 (10)	0.0007 (9)	0.0020 (10)
C1	0.0190 (13)	0.0262 (14)	0.0259 (13)	0.0022 (12)	−0.0040 (11)	0.0008 (11)
C2	0.0242 (14)	0.0292 (15)	0.0250 (14)	0.0024 (12)	0.0000 (11)	0.0011 (11)
C3	0.0239 (14)	0.0315 (16)	0.0277 (14)	0.0011 (12)	−0.0007 (11)	−0.0021 (12)
C4	0.0236 (13)	0.0310 (15)	0.0250 (13)	−0.0040 (12)	−0.0002 (11)	0.0021 (11)
C5	0.0173 (12)	0.0276 (14)	0.0260 (13)	−0.0033 (11)	−0.0014 (10)	−0.0003 (11)
C6	0.0205 (13)	0.0237 (14)	0.0290 (13)	−0.0048 (11)	0.0002 (12)	0.0031 (11)
C7	0.0286 (15)	0.0290 (15)	0.0342 (15)	0.0004 (12)	0.0018 (12)	−0.0032 (12)
C8	0.0282 (15)	0.0328 (15)	0.0297 (14)	−0.0045 (13)	0.0040 (12)	−0.0040 (11)
C9	0.0241 (14)	0.0308 (15)	0.0251 (13)	−0.0072 (12)	−0.0008 (11)	0.0014 (11)
C10	0.0203 (13)	0.0284 (15)	0.0295 (14)	−0.0005 (11)	−0.0008 (11)	−0.0008 (11)
C11	0.0282 (14)	0.0354 (16)	0.0250 (14)	−0.0024 (13)	−0.0027 (11)	0.0050 (12)
C12	0.0562 (19)	0.0416 (18)	0.0292 (15)	−0.0041 (15)	−0.0075 (14)	0.0021 (14)
C13	0.066 (2)	0.058 (2)	0.0279 (15)	−0.0043 (18)	−0.0083 (14)	0.0001 (15)
C14	0.055 (2)	0.063 (2)	0.0325 (17)	−0.0007 (17)	−0.0086 (15)	0.0163 (15)
C15	0.0481 (19)	0.050 (2)	0.0462 (18)	0.0091 (16)	−0.0025 (15)	0.0125 (15)
C16	0.0401 (17)	0.0511 (19)	0.0300 (15)	0.0032 (15)	−0.0031 (13)	0.0007 (13)
C17	0.0339 (15)	0.0339 (16)	0.0299 (13)	0.0007 (13)	−0.0056 (12)	−0.0002 (11)
C18	0.0368 (16)	0.0435 (17)	0.0310 (14)	0.0005 (14)	0.0038 (12)	−0.0007 (13)

### Geometric parameters (Å, °)

**Table:**

O1—C6	1.358 (2)	C7—C8	1.375 (3)
O1—H1A	1.01 (2)	C7—H7	0.9300
N1—N2	1.265 (3)	C8—C9	1.387 (3)
N1—C11	1.429 (3)	C8—H8	0.9300
N2—C9	1.422 (3)	C9—C10	1.401 (3)
N3—C2	1.482 (3)	C10—H10	0.9300
N3—C1	1.483 (3)	C11—C12	1.380 (3)
N3—H3A	0.92 (2)	C11—C16	1.384 (3)
N4—C1	1.451 (3)	C12—C13	1.380 (3)
N4—C3	1.473 (3)	C12—H12	0.9300
N4—H4A	0.93 (2)	C13—C14	1.377 (4)
C1—C5	1.518 (3)	C13—H13	0.9300
C1—H1	1.04 (2)	C14—C15	1.374 (3)
C2—C4	1.525 (3)	C14—H14	0.9300
C2—H21	1.05 (2)	C15—C16	1.385 (3)
C2—H22	1.03 (2)	C15—H15	0.9300
C3—C4	1.537 (3)	C16—H16	0.9300
C3—H31	1.03 (2)	C17—H17A	0.9600
C3—H32	0.98 (2)	C17—H17B	0.9600
C4—C17	1.518 (3)	C17—H17C	0.9600
C4—C18	1.531 (3)	C18—H18A	0.9600
C5—C10	1.375 (3)	C18—H18B	0.9600
C5—C6	1.412 (3)	C18—H18C	0.9600
C6—C7	1.385 (3)		
			
C6—O1—H1A	104.5 (13)	C6—C7—H7	120.1
N2—N1—C11	114.21 (19)	C7—C8—C9	120.5 (2)
N1—N2—C9	114.67 (19)	C7—C8—H8	119.7
C2—N3—C1	110.27 (18)	C9—C8—H8	119.7
C2—N3—H3A	108.7 (14)	C8—C9—C10	119.4 (2)
C1—N3—H3A	107.3 (14)	C8—C9—N2	115.7 (2)
C1—N4—C3	111.13 (19)	C10—C9—N2	124.8 (2)
C1—N4—H4A	113.1 (14)	C5—C10—C9	120.9 (2)
C3—N4—H4A	104.4 (14)	C5—C10—H10	119.5
N4—C1—N3	115.15 (19)	C9—C10—H10	119.5
N4—C1—C5	112.51 (19)	C12—C11—C16	119.7 (2)
N3—C1—C5	109.76 (18)	C12—C11—N1	124.5 (2)
N4—C1—H1	106.2 (12)	C16—C11—N1	115.7 (2)
N3—C1—H1	106.5 (12)	C11—C12—C13	119.6 (3)
C5—C1—H1	106.1 (11)	C11—C12—H12	120.2
N3—C2—C4	113.06 (19)	C13—C12—H12	120.2
N3—C2—H21	103.4 (12)	C14—C13—C12	120.6 (2)
C4—C2—H21	110.0 (13)	C14—C13—H13	119.7
N3—C2—H22	107.0 (12)	C12—C13—H13	119.7
C4—C2—H22	112.2 (13)	C15—C14—C13	120.0 (3)
H21—C2—H22	110.8 (17)	C15—C14—H14	120.0
N4—C3—C4	115.46 (19)	C13—C14—H14	120.0
N4—C3—H31	105.8 (12)	C14—C15—C16	119.8 (3)
C4—C3—H31	108.8 (12)	C14—C15—H15	120.1
N4—C3—H32	108.5 (13)	C16—C15—H15	120.1
C4—C3—H32	109.2 (13)	C11—C16—C15	120.3 (2)
H31—C3—H32	108.9 (18)	C11—C16—H16	119.9
C17—C4—C2	110.9 (2)	C15—C16—H16	119.9
C17—C4—C18	109.10 (19)	C4—C17—H17A	109.5
C2—C4—C18	109.10 (19)	C4—C17—H17B	109.5
C17—C4—C3	111.07 (19)	H17A—C17—H17B	109.5
C2—C4—C3	108.18 (19)	C4—C17—H17C	109.5
C18—C4—C3	108.39 (19)	H17A—C17—H17C	109.5
C10—C5—C6	118.6 (2)	H17B—C17—H17C	109.5
C10—C5—C1	122.9 (2)	C4—C18—H18A	109.5
C6—C5—C1	118.5 (2)	C4—C18—H18B	109.5
O1—C6—C7	118.7 (2)	H18A—C18—H18B	109.5
O1—C6—C5	120.7 (2)	C4—C18—H18C	109.5
C7—C6—C5	120.6 (2)	H18A—C18—H18C	109.5
C8—C7—C6	119.9 (2)	H18B—C18—H18C	109.5
C8—C7—H7	120.1		
			
C11—N1—N2—C9	179.73 (19)	O1—C6—C7—C8	179.6 (2)
C3—N4—C1—N3	52.1 (3)	C5—C6—C7—C8	−0.4 (3)
C3—N4—C1—C5	178.90 (19)	C6—C7—C8—C9	−1.2 (4)
C2—N3—C1—N4	−55.2 (3)	C7—C8—C9—C10	2.1 (3)
C2—N3—C1—C5	176.64 (19)	C7—C8—C9—N2	−178.7 (2)
C1—N3—C2—C4	55.4 (3)	N1—N2—C9—C8	173.0 (2)
C1—N4—C3—C4	−50.2 (3)	N1—N2—C9—C10	−7.9 (3)
N3—C2—C4—C17	69.7 (2)	C6—C5—C10—C9	−0.1 (3)
N3—C2—C4—C18	−170.09 (19)	C1—C5—C10—C9	177.5 (2)
N3—C2—C4—C3	−52.4 (3)	C8—C9—C10—C5	−1.4 (3)
N4—C3—C4—C17	−71.9 (3)	N2—C9—C10—C5	179.4 (2)
N4—C3—C4—C2	50.1 (3)	N2—N1—C11—C12	−7.3 (3)
N4—C3—C4—C18	168.3 (2)	N2—N1—C11—C16	171.9 (2)
N4—C1—C5—C10	10.1 (3)	C16—C11—C12—C13	0.0 (4)
N3—C1—C5—C10	139.7 (2)	N1—C11—C12—C13	179.2 (2)
N4—C1—C5—C6	−172.34 (19)	C11—C12—C13—C14	0.0 (4)
N3—C1—C5—C6	−42.7 (3)	C12—C13—C14—C15	−0.3 (4)
C10—C5—C6—O1	−178.9 (2)	C13—C14—C15—C16	0.6 (4)
C1—C5—C6—O1	3.4 (3)	C12—C11—C16—C15	0.3 (4)
C10—C5—C6—C7	1.0 (3)	N1—C11—C16—C15	−179.0 (2)
C1—C5—C6—C7	−176.7 (2)	C14—C15—C16—C11	−0.6 (4)

### Hydrogen-bond geometry (Å, °)

**Table:**

*D*—H···*A*	*D*—H	H···*A*	*D*···*A*	*D*—H···*A*
O1—H1A···N3	1.01 (2)	1.65 (2)	2.584 (2)	152 (2)
N3—H3A···N4^i^	0.92 (2)	2.30 (2)	3.159 (3)	155 (2)
N4—H4A···O1^ii^	0.93 (2)	2.18 (2)	3.106 (3)	172 (2)

Symmetry codes: (i) *x*−1/2, *y*, −*z*+1/2; (ii) −*x*+3/2, *y*−1/2, *z*.
